# New Bright Luminescent Metal–Organic Frameworks Based on Heterometallic Gadolinium and Terbium Chloroterephthalates for Fingerprinting and Heavy-Metal Detection

**DOI:** 10.3390/molecules31162834

**Published:** 2026-08-14

**Authors:** Oleg S. Butorlin, Anna S. Petrova, Aleksei E. Mikhaltsov, Mikhail N. Ryazantsev, Nikita A. Bogachev, Mikhail Yu. Skripkin, Andrey S. Mereshchenko

**Affiliations:** 1 Institution of Chemistry, Saint-Petersburg State University, 199034 St. Petersburg, Russia; olbuse@mail.ru (O.S.B.); an.petra04.floreo@gmail.com (A.S.P.); st112377@student.spbu.ru (A.E.M.); mikhail.n.ryazantsev@gmail.com (M.N.R.); n.bogachev@spbu.ru (N.A.B.); skripkin1965@yandex.ru (M.Y.S.); 2 Institute of Biomedical Systems and Biotechnologies, Peter the Great St. Petersburg Polytechnic University, 195251 St. Petersburg, Russia; 3 School of Physical Sciences and Nanotechnology, Yachay Tech University, Urcuquí 100115, Ecuador

**Keywords:** metal–organic framework, luminescence, rare earth, terbium, gadolinium, antenna effect, heavy-metal detection, fingerprinting

## Abstract

A series of novel heterometallic rare-earth chloroterephthalate metal–organic frameworks with the general formula (Tb_x_Gd_1−x_)_2_(Cl-1,4-bdc)_3_·5H_2_O (x = 0–1) were synthesized via direct precipitation from aqueous solutions. The structural and photophysical properties of these compounds were studied in detail. All compounds exhibit bright luminescence upon UV excitation into the ligand absorption band due to an efficient antenna effect. The photoluminescence quantum yield shows a non-monotonic dependence on the concentration of the terbium ion with a maximum value of 71% achieved for the compound containing equal molar fractions of the lanthanide ions. The (Tb_0.5_Gd_0.5_)_2_(Cl-1,4-bdc)_3_·5H_2_O sample was evaluatedfor its utility in both qualitative and quantitative analysis of selected metal ions and in latent fingerprint development. It was shown to enable the detection of Cr(III), Fe(III), and Cu(II) ions through luminescence quenching, with the emission intensity being concentration-dependent. This behaviour highlights the compound’s potential as a basis for analytical protocols and materials aimed at the quantitative determination of these metal ions.

## 1. Introduction

Metal–organic frameworks (MOFs) have attracted considerable attention from the scientific community in recent years. The most vivid confirmation of this fact can be considered the Nobel Prize in Chemistry of 2025. Owing to their multifunctionality, MOFs find numerous applications ranging from chemistry and medicine to quantum technologies and forensic science. Among them, luminescent MOFs occupy a special place, in which rare-earth element (REE) ions serve as metal nodes. These compounds exhibit unique optical properties arising from f–f transitions of the REE ions, including narrow spectral lines, long excited state lifetimes (up to milliseconds), minimal sensitivity of the emission to the external environment (except for certain hypersensitive transitions), and high colour purity. These characteristics make REE-MOFs promising candidates for applications in organic light-emitting diodes [1,2], luminescent sensors [3,4], materials for bioimaging [5,6] and medicine [7,8], optical thermometers [9,10], photovoltaic systems [11,12], and other fields [13,14,15]. However, direct excitation of REE ions is inefficient due to the forbidden nature of f–f transitions, which results in low absorption coefficients and weak photoluminescence in inorganic REE compounds. Developing highly efficient materials requires overcoming this limitation. Additional factors that reduce emission intensity include concentration quenching (self-quenching) due to energy transfer between closely spaced active ions [16,17] and non-radiative relaxation through vibrations of O-H and C-H bonds of coordinated solvent molecules [18,19,20]. The latter issue can be addressed by replacing the solvent [21,22] and/or drying of compounds until complete removal of coordinated solvent [23]. The problem of self-quenching is often solved by introducing optically inactive REE ions (Gd^3+^, La^3+^, Y^3+^, Lu^3+^) into the crystalline matrix without disrupting the structure [24,25,26]. The forbidden nature of f–f transitions is often circumvented by the low local symmetry of REE ions in most of their compounds [15,25,27,28,29,30]. Compounds with high site symmetry generally exhibit lower quantum yields than their low-symmetry counterparts [31,32,33]. In this context, a widely adopted strategy involves the use of specially designed ligands that can efficiently absorb light and transfer the excitation energy to the metal ion. Following photon absorption, intersystem crossing populates the ligand triplet state (T_1_), which is then followed by energy transfer to an excited level of the REE ion. This mechanism is known as the antenna effect [34,35,36]. The efficiency of this process critically depends on the energy gap (ΔE) between the donor excited state (typically T_1_) of the ligand and the acceptor level of the REE ion. The optimal ΔE is generally considered to lie in the range of ΔE = 2500–3500 cm^−1^ [37]. An important means of tuning the T_1_ energy is chemical modification of the ligand. For example, introducing electron-withdrawing substituents such as halogens into the aromatic core of the ligand can lower the T_1_ energy, thereby adjusting it for optimal energy transfer to a specific REE ion [38,39]. Aromatic dicarboxylates, particularly terephthalates, rank among the most extensively studied antenna linkers for the development of luminescent REE-MOFs [30,40,41,42,43]. While unsubstituted REE terephthalates have been investigated in considerable detail, their halogenated derivatives have received substantially less attention. Most studies have focused on heavy halogens (iodine and bromine), owing to their pronounced influence on the supramolecular organization of the structures through halogen bonding [44,45] and the heavy-atom effect, which enhances intersystem crossing and the efficiency of REE ion luminescence [46,47,48]. Thus, the luminescence of lanthanide diiodo-, tetraiodo-, and tetrabromoterephthalates has been reported; however, a systematic analysis of the quantum yield dependence on the REE ion concentration has generally not been reported in these studiesin these studies [49,50].

Chloro-substituted terephthalates have also attracted attention, but investigation of them has followed a different direction. Several studies have addressed the structural chemistry of polychloroterephthalates of d-metals [51,52,53,54]; for REEs, such reports remain scarce [55]. Consequently, monochloroterephthalates as a class of ligands have received comparatively little attention. The available publications are mostly focused on their use in transition metal MOFs [56,57]. Comprehensive studies on the photophysical and structural properties of REE monochloroterephthalates are virtually absent from the literature.

In the present work, we studied in detail the structural and photophysical properties of a series of terbium–gadolinium monochloroterephthalate compounds over a wide range of luminescent ion concentrations, aiming to identify the composition with the highest quantum yield. We also demonstrate the potential of these materials for applications in analytical chemistry and fingerprinting.

## 2. Results and Discussion

### 2.1. Composition of Compounds

**Figure 1:**
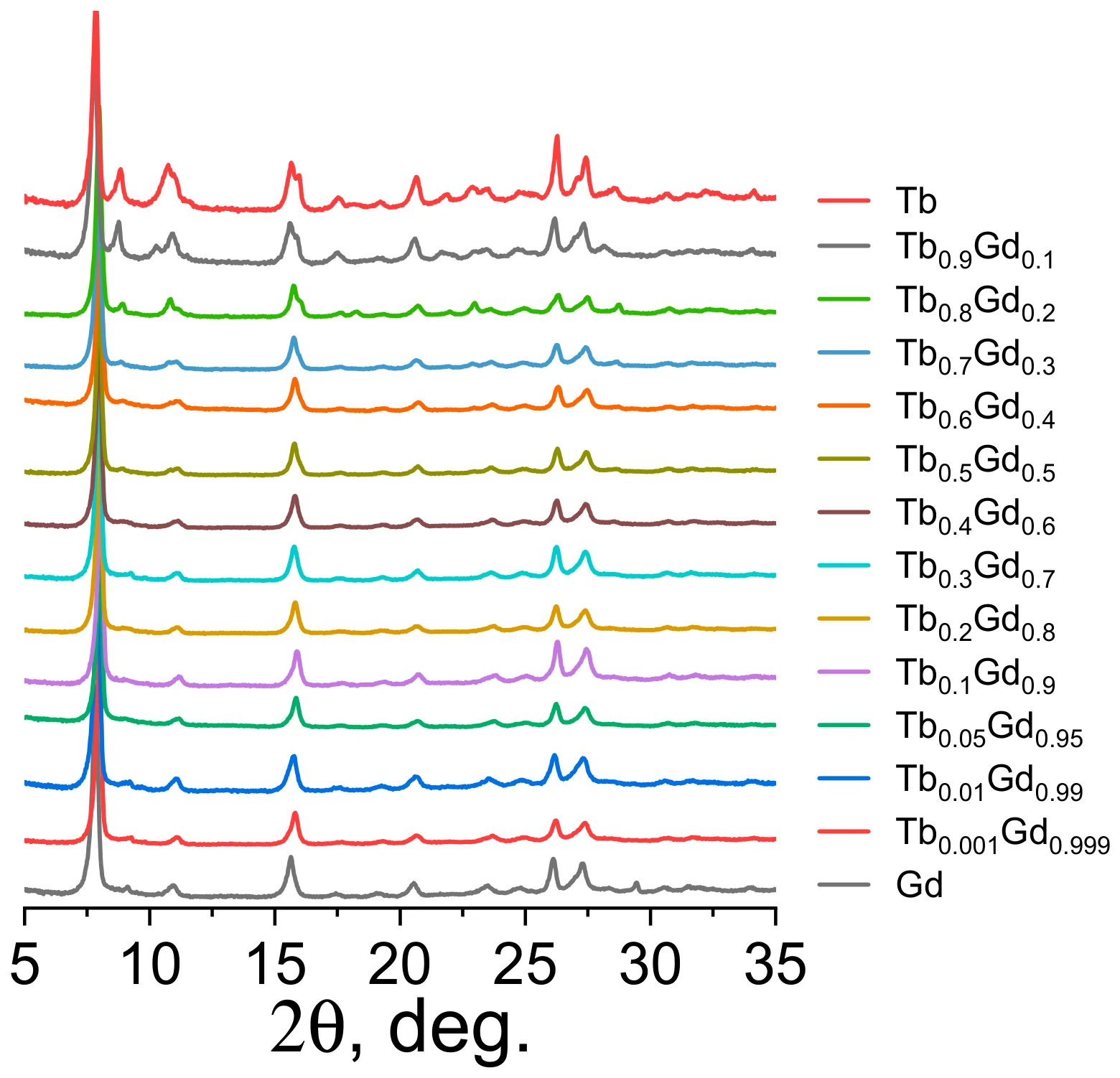
Powder diffraction patterns of compounds of the series (Tb_x_Gd_1−x_)_2_(Cl-1,4-bdc)_3_∙5H_2_O (x = 0–1).

The powder X-ray diffraction (PXRD) patterns of the synthesized compounds are shown in Figure 1. The PXRD data indicate that the structure of the chloroterephthalate complexes is invariant with respect to the Tb/Gd ratio across the series, confirming the formation of solid solutions.

**Figure 2:**
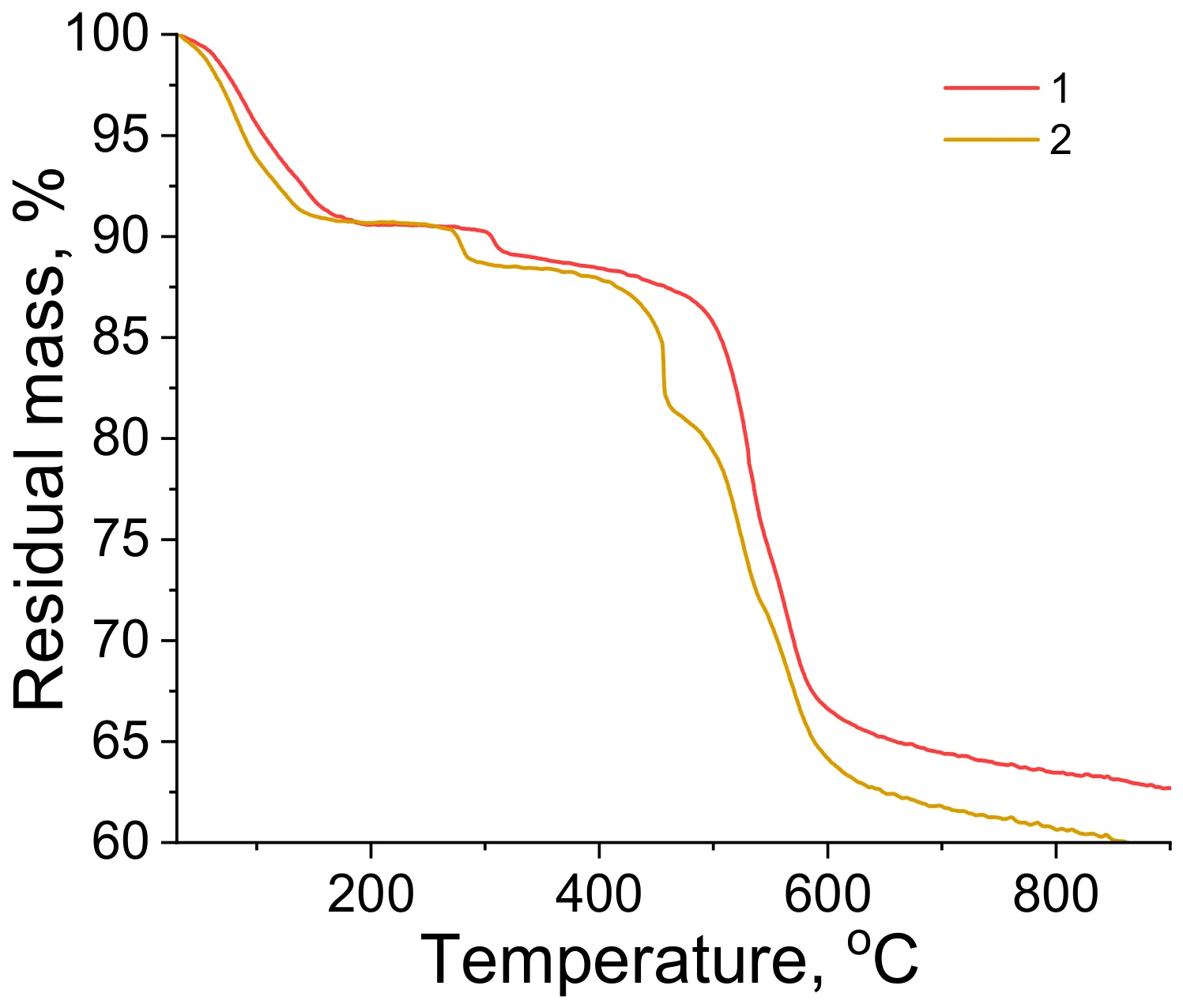
Thermogravimetric analysis curves for terbium (1) and gadolinium (2) compounds.

**Figure 3:**
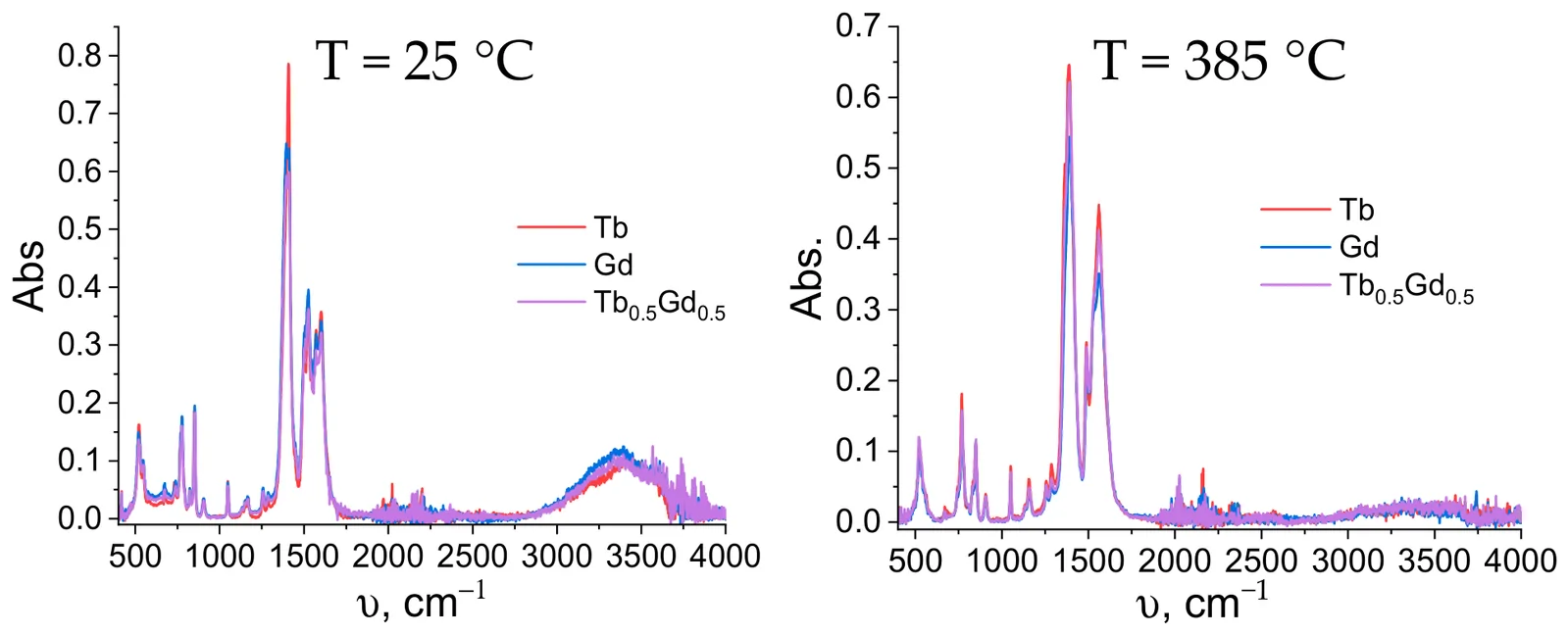
FTIR spectra of neat Tb_2_(Cl-1,4-bdc)_3_∙5H_2_O, Gd_2_(Cl-1,4-bdc)_3_∙5H_2_O and of heterometallic (Tb_0.5_Gd_0.5_)_2_(Cl-1,4-bdc)_3_∙5H_2_O, measured after heating at temperatures of 25 and 385 °C.

The composition of as-synthesized compounds was determined by thermogravimetric analysis (TGA), elemental analysis, energy dispersive X-ray spectroscopy (EDX), infrared (IR) spectroscopy, and variable-temperature powder X-ray diffraction (VT-PXRD). The EDX results (Table 1) showed that the metal ratios in the compounds correspond to the expected stoichiometric values. Thermogravimetric analysis (Figure 2) reveals several decomposition processes occurring at around 130–150 °C and 280–310 °C, which, most probably, correspond to the loss of water molecules. For the chloro-derivatives several decomposition processes occur at around 130–150 °C and 280-310 °C accompanied with mass loss 9.2–9.4 wt% that correspond to 5.2–5.3 water molecules per formula unit. This assumption was confirmed by the IR spectroscopy of the selected compounds, namely (Tb_x_Gd_1−x_)_2_(Cl-1,4-bdc)_3_∙5H_2_O (x = 0, 0.5, 1), before and after calcination at of 385 °C (Figure 3). The broad band centred around 3500 cm^−1^ is attributed to the O–H stretching vibrations of water molecules. The series of sharp bands observed in the 1270–1470 and 1470–1800 cm^−1^ ranges are assigned to the symmetric and asymmetric stretching modes, respectively, of the carboxylate (–COO^−^) groups in the chloroterephthalate ion [58,59]. One can observe that after calcination, the intensity of the 3500 cm^−1^ band significantly decreases and almost vanishes after 385 °C calcination, whereas the 1270–1470 and 1470–1800 cm^−1^ bands remain almost unchanged, which shows that the heating of these compounds up to 385 °C results in dehydration, whereas the organic ligand remains unchanged. In addition, the spectra exhibit bands in the range 600–800 cm^−1^, which are assigned to C–Cl stretching vibrations, and multiple peaks between 1400 and 1600 cm^−1^ corresponding to the aromatic ring skeletal vibrations. The C–H out-of-plane bending modes of the aromatic ring appear as weak bands near 750–850 cm^−1^.

The elemental analysis (Table 2) was additionally used to determine the number of crystallization water molecules in the halogenoterephthalates. The theoretical and experimental values given in Table 2 converge within the method’s error margin under the assumption that the chloro-derivatives contain 5 molecules of water of crystallization per formula unit. The IR spectra (Figure 3) of the chloroterephthalates, which are thermally stable up to higher temperatures, were recorded after heating at 385 °C. The disappearance of the band at 3300–3500 cm^−1^ indicates the removal of water and confirms that the observed mass loss originates from the release of water of crystallization and not from degradation of the organic framework.

Thus, based on TGA, powder X-ray diffraction analysis, IR spectroscopy, and elemental analysis, the composition and the number of crystallization water molecules were determined for the obtained series of compounds: (Tb_x_Gd_1−x_)_2_(Cl-1,4-bdc)_3_·5H_2_O. The structures of the obtained compounds are similar to each other, a conclusion drawn from two cross-referenced factors: the matching powder X-ray diffraction patterns and the matching fine structure of the bands in the IR spectra of the compounds.

**Figure 4:**
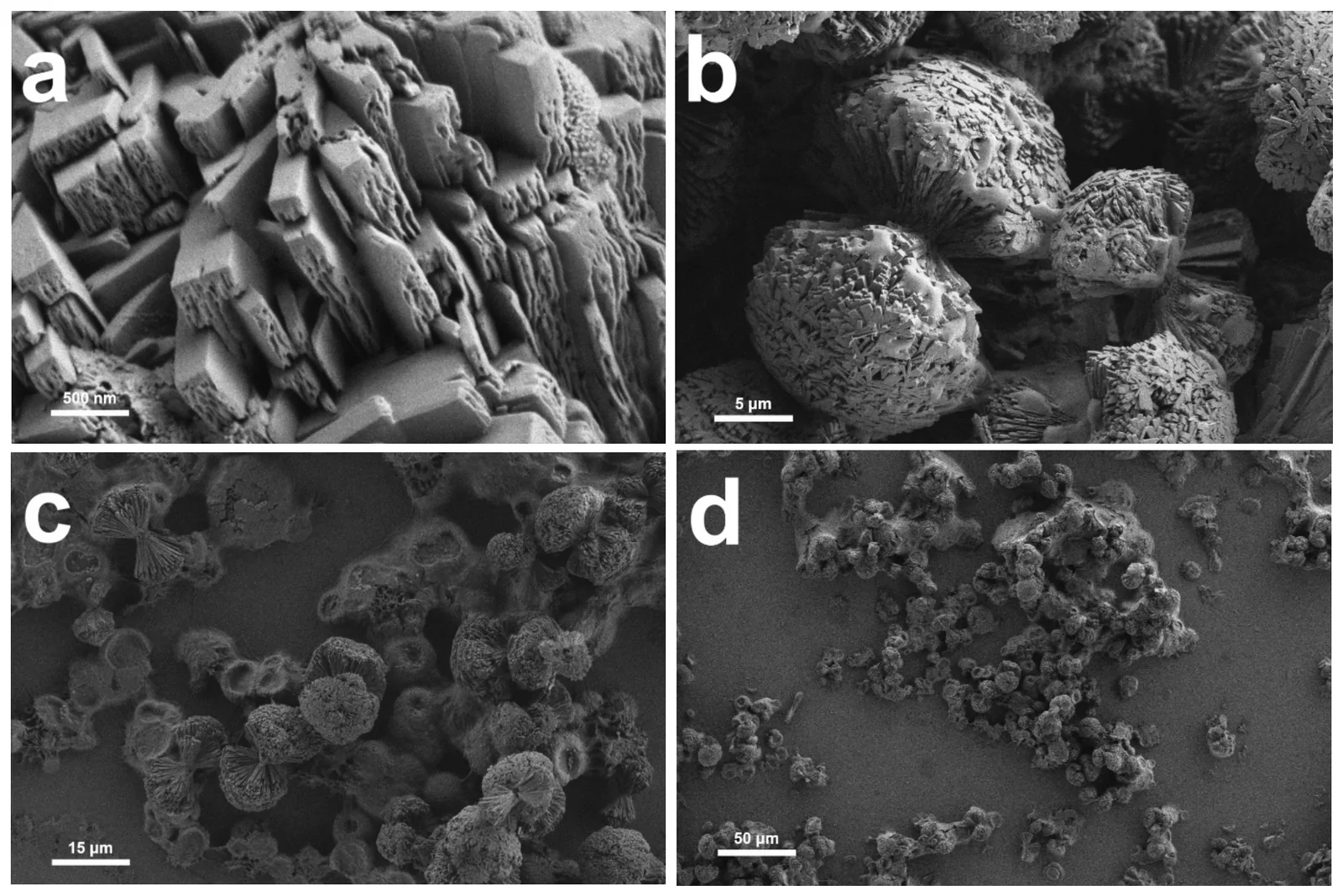
SEM images of Tb_2_(Cl-1,4-bdc)_3_∙5H_2_O samples: (**a**) magnification 30000:1 with the 500 nm scale bar (s.b.); (**b**) 3000:1 with the 5 μm s.b.; (**c**) 1000:1 with the 15 μm s.b.; (**d**) 300:1 with the 50 μm s.b.

The morphology of the particles of the selected synthesized compounds was determined by scanning electron microscopy (SEM). In all samples, particles with an hourglass shape are formed (average diameter d_av._ = 15–30 μm). Hemispherical particles (d_av._ = 8–20 μm) begin to grow simultaneously from a single nucleation centre in opposite directions (Figure 4b–d). The growing halves of the particles consist of plate-like aggregates with varying thicknesses (30–400 nm). Analysis of the obtained images shows that the particle morphology does not depend on the composition of the compounds, which is consistent with the results of other analyses (XRD, TGA, IR) and confirms that the structure and phase composition are independent of the chemical composition.

### 2.2. Luminescence

**Figure 5:**
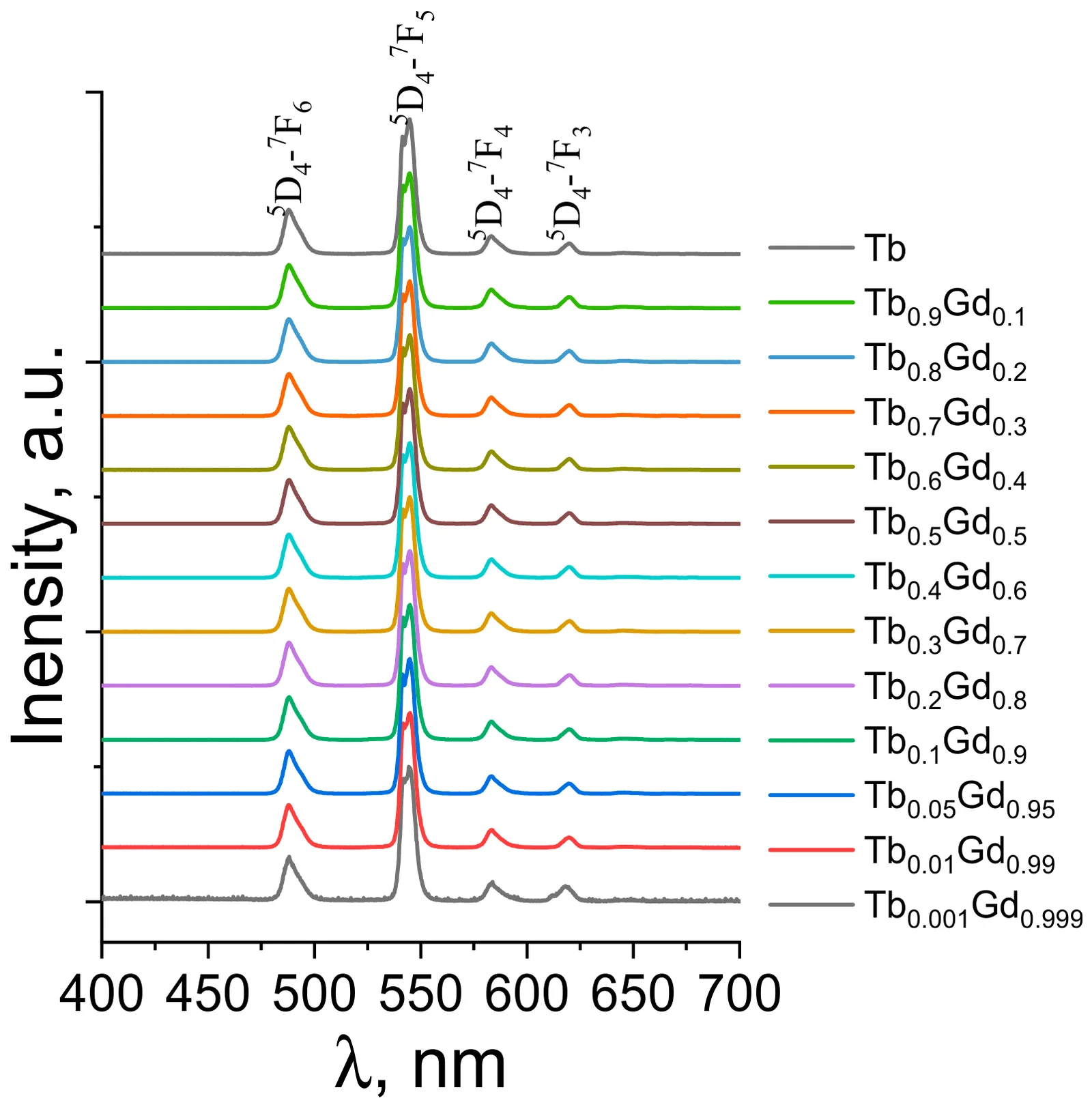
Emission spectra of (Tb_x_Gd_1−x_)_2_(Cl-1,4-bdc)_3_∙5H_2_O (x = 0–1).

**Figure 6:**
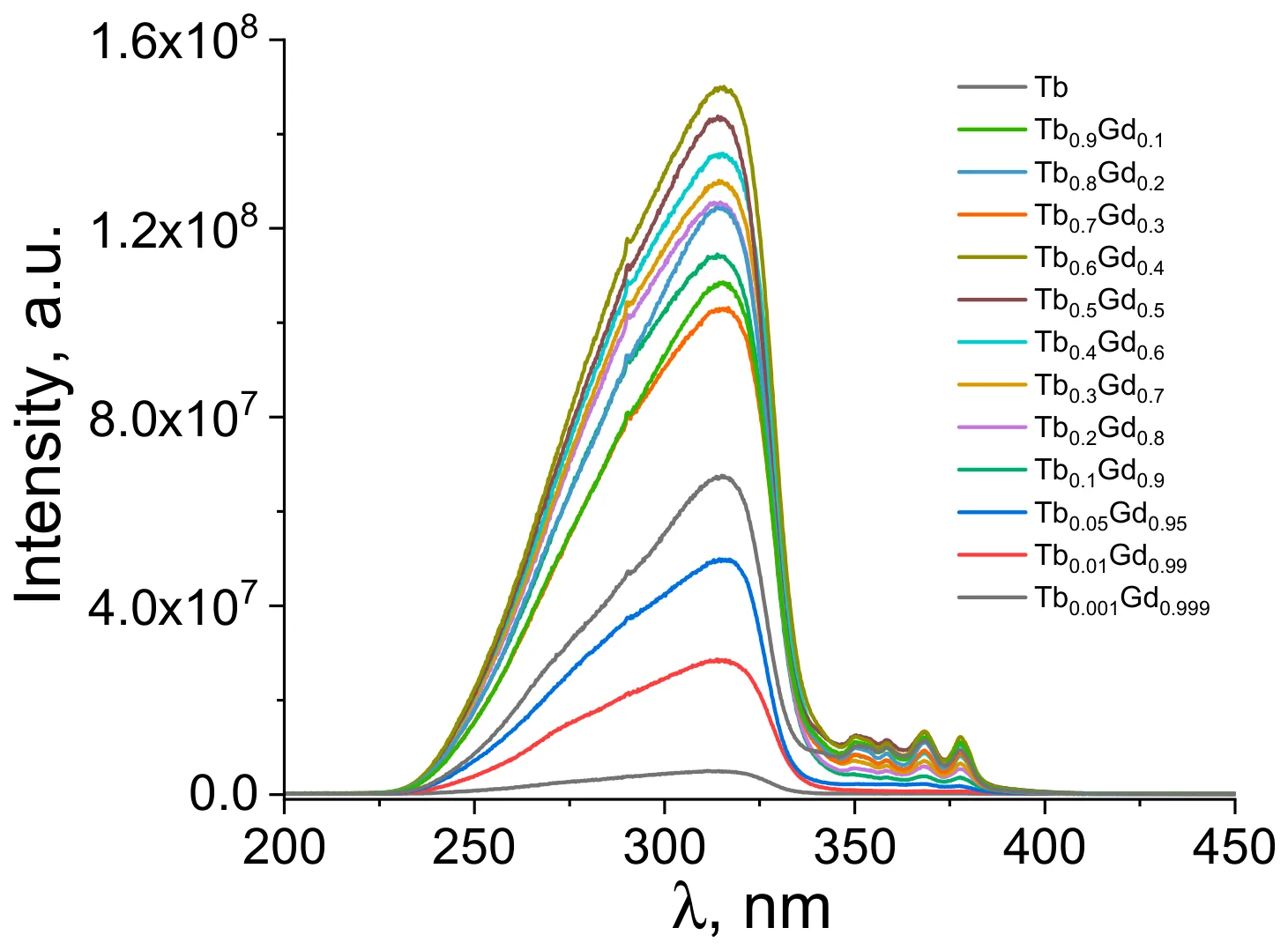
Photoluminescence excitation spectra of (Tb_x_Gd_1−x_)_2_(Cl-1,4-bdc)_3_∙5H_2_O (x = 0–1).

Figure 5 presents a comparison of the emission spectra for the investigated series of terbium-gadolinium chloroterephthalate MOFs, (Tb_x_Gd_1−x_)_2_(Cl-1,4-bdc)_3_∙5H_2_O (x = 0–1), upon 320 nm excitation into the absorption band of the terephthalate ion. The excitation wavelength was selected based on the luminescence excitation spectra (Figure 6) monitored at emission wavelengths of 544 nm (corresponding to their luminescence intensity maxima). The excitation spectra contain a broad band corresponding to the absorption of the terephthalate ion. In addition to the ligand-centred band, weak features are observed above 320 nm, which can be attributed to direct f–f transitions of Tb^3+^ ions (e.g., ^7^F_6_ → ^5^D_4_). These minor contributions do not affect the overall sensitization mechanism but are typical for concentrated terbium compounds. In the emission spectra, there are f-f transition bands of the lanthanide ion. This indicates the presence of an antenna effect (sensitized luminescence) in all the studied compounds. The emission bands in the spectra of the terbium compounds correspond to the following f-f transitions of the Tb^3+^ ion: ^5^D_4_ → ^7^F_6_ (488 nm), ^5^D_4_ → ^7^F_5_ (544 nm, highest intensity), ^5^D_4_ → ^7^F_4_ (583 nm), ^5^D_4_ → ^7^F_3_ (620 nm). This further confirms the X-ray phase analysis results regarding the uniform phase composition of the obtained compounds.

**Figure 7:**
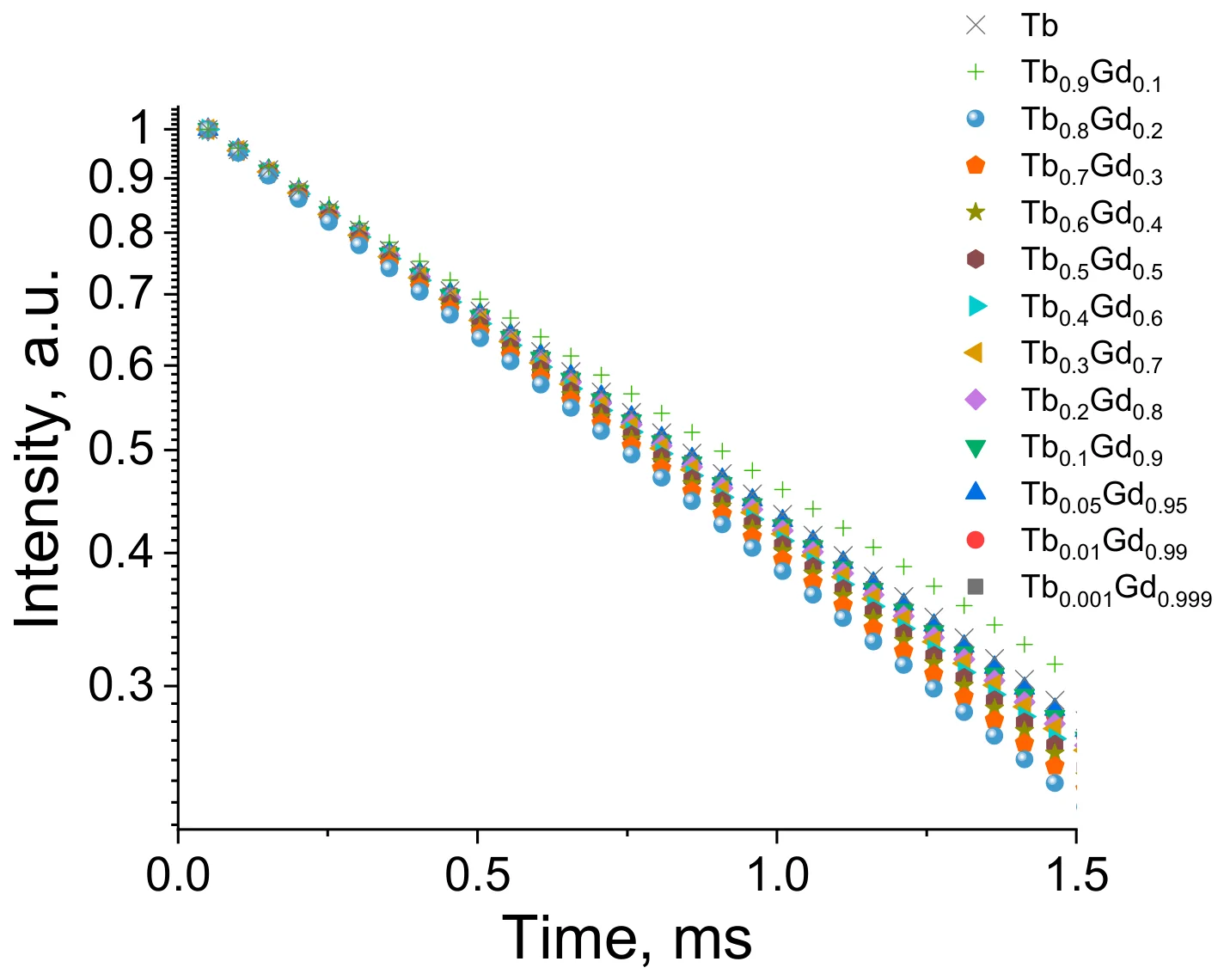
Photoluminescence decay curves for the series (Tb_x_Gd_1−x_)_2_(Cl-1,4-bdc)_3_∙5H_2_O.

The photoluminescence decay kinetics curves for the synthesized compounds are presented in Figure 7. The curves were fitted by monoexponential decay function:

$$I\left(t\right)=I_{0}e^{-\frac{t}{\tau }}$$

**Figure 8:**
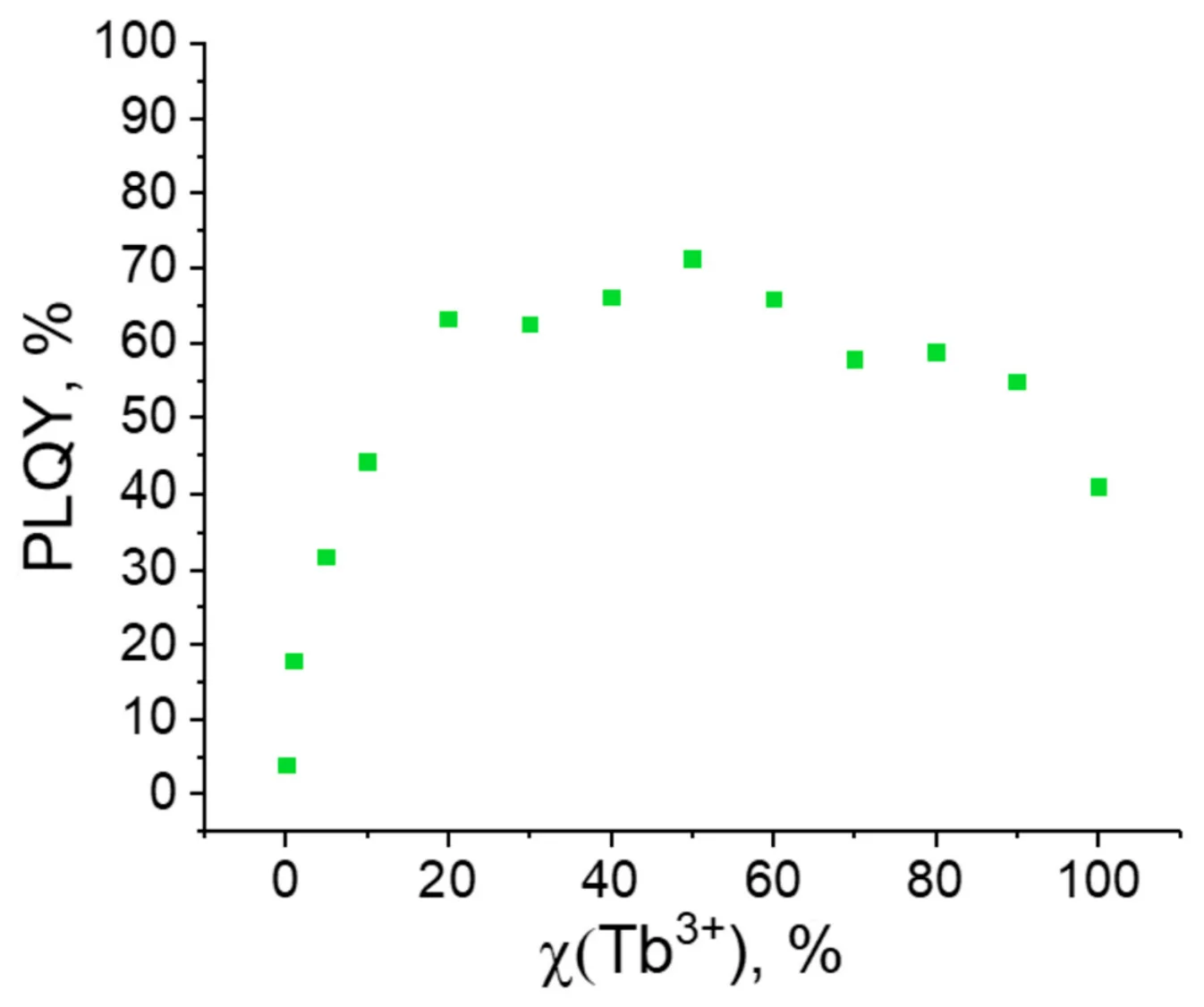
Dependence of PLQYs on the composition of (Tb_x_Gd_1−x_)_2_(Cl-1,4-bdc)_3_∙5H_2_O samples (x = 0–1).

Based on the analysis of the luminescence decay curves, the lifetimes of the excited state (^5^D_4_) of the Tb^3+^ were determined (Figure 8; Table 3).

The lifetimes of the excited states of the luminescent ions gradually decrease with an increase in the concentration of these ions and a decrease in the concentration of Gd^3+^ ions. These results are consistent with our previous studies [24,25]; we attribute this phenomenon to a higher probability of energy transfer between closely spaced Tb^3+^ ions, as well as to quenching by impurities at higher concentrations of the luminescent ions. The following compound possesses the maximum value of quantum yield and luminescence intensity: (Tb_0.5_Gd_0.5_)_2_(Cl-1,4-bdc)_3_·5H_2_O (71 ± 1%). The plot of quantum yield dependence on the terbium mole fraction shows an initial gradual increase in intensity, associated with the increase in concentration of the luminescent ion (Tb^3+^), followed by a gradual decrease, which is attributed tothe dissipation of accumulated energy into thermal vibrations and self-quenching of the luminescent centres (concentration quenching).

Thus, the analysis of the photophysical properties of MOFs based on heterometallic terbium chloroterephthalates allowed us to identify the compositions exhibiting the most intense luminescence and highest quantum yields.

## 3. Application of (Tb_0.5_Gd_0.5_)_2_(Cl-1,4-bdc)_3_·5H_2_O

### 3.1. Heavy-Metal Detection

**Figure 9:**
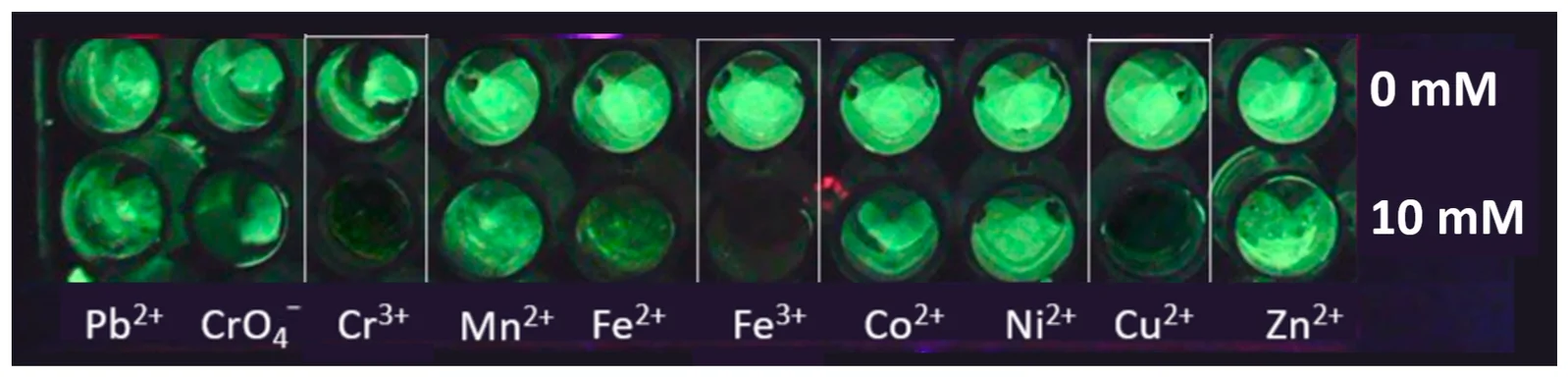
The influence of different ions on luminescence (Tb_0.5_Gd_0.5_)_2_(Cl-1,4-bdc)_3_·5H_2_O.

The effect of various ions in solution on the luminescent properties of (Tb_0.5_Gd_0.5_)_2_(Cl-1,4-bdc)_3_·5H_2_O, as the sample with the highest luminescence quantum yield, was studied. Prior to dosing, the MOF suspensions were subjected to ultrasonic treatment for 10 min in order to ensure complete deagglomeration and uniform dispersion. During the process of aliquoting, continuous agitation of the bulk suspension was maintained in order to ensure a uniform solid–liquid distribution and prevent any particle settling. First, screening was performed to identify ions that cause luminescence quenching. Aliquots of 20 µL of a suspension containing 4 × 10^−6^ mol of the luminescent compound were dispensed into the wells of a 96-well plate. To obtain the control samples (Figure 9, top row), 200 µL of distilled water was added to the suspension. For the test samples, 200 µL of a 10 mM solution of each salt (nitrates for metal cations, or the sodium salt for chromate) containing one of the following ions was added: Pb^2+^, CrO_4_^2−^, Cr^3+^, Mn^2+^, Fe^2+^, Fe^3+^, Co^2+^, Ni^2+^, Cu^2+^, or Zn^2+^ (Figure 9, bottom row). The solutions in the wells were dried at 60 °C for 12 h. The plates were then placed in a UV chamber (254 nm), and photographs of the luminescent samples were taken under UV illumination (Figure 9). The (Tb_0.5_Gd_0.5_)_2_(Cl-1,4-bdc)_3_·5H_2_O samples emit green light, and addition of heavy metal ions decreses the intensity of emission (quenches the photoluminescence). The small red spots correspond to artefact reflection from 96-well plate and do not affect on the results.

The screening results revealed that, among the ten tested species, only Cr^3+^, Fe^3+^, and Cu^2+^ cause significant luminescence quenching. To address potential concerns regarding salt hydrolysis and pH effects, a control experiment was conducted by holding the MOF in solutions at a pH of 1.5 and above for 12 h, followed by an ICP analysis for the content of lanthanide ions. It showed that when holding the solution at this pH, the concentration of lanthanide ions does not increase compared to the initial solution, which indicates the absence of destruction of these MOFs.

**Figure 10:**
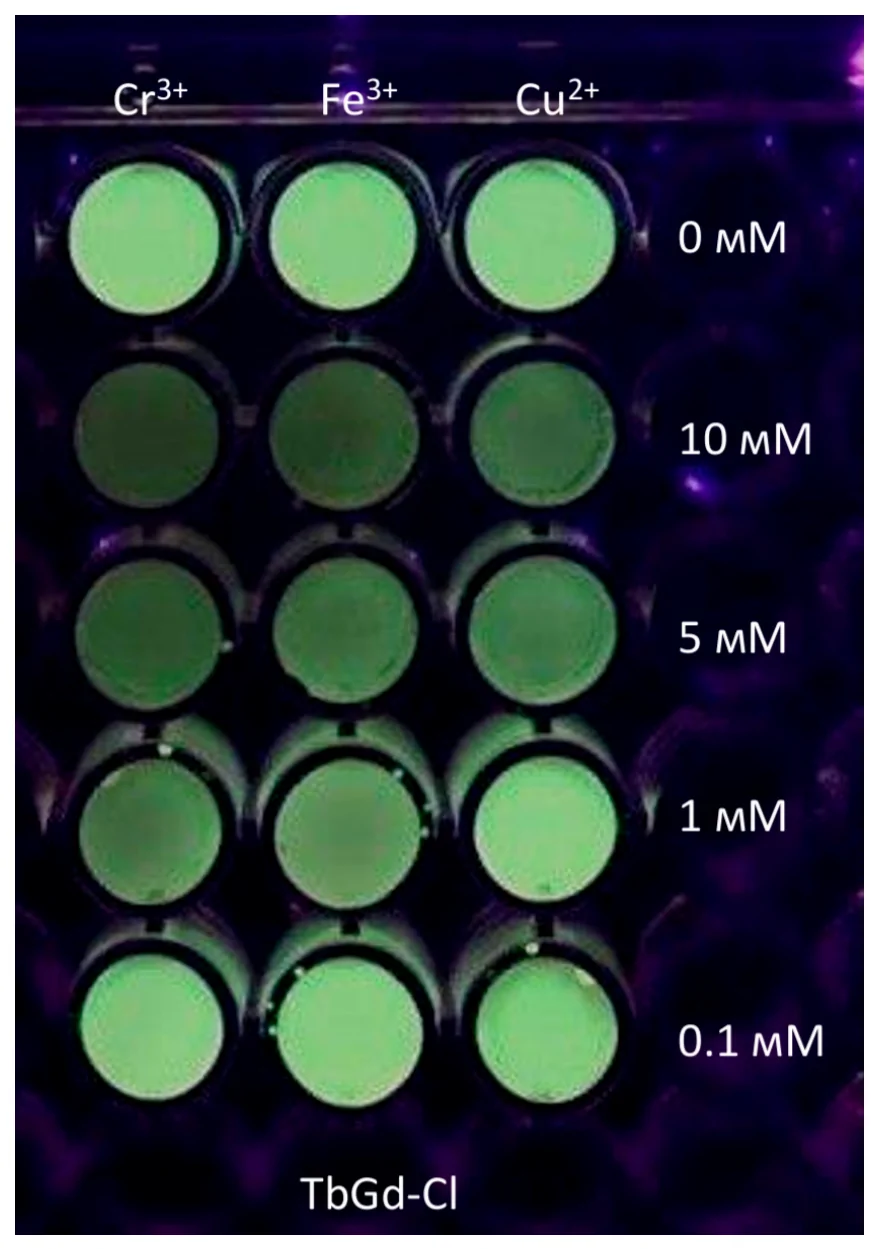
Screening results with different concentrations of metal ions.

To evaluate the sensitivity of the synthesized compounds to these quenchers, the luminescence intensity was measured at various concentrations of the corresponding metal salts. For this purpose, different amounts of each salt were added to separate sets of solutions to obtain final concentrations in the range of 0.1 to 10 mM, along with a control sample containing only distilled water. The solutions in the wells were dried at 60 °C for 12 h. The plates were then placed in a UV chamber (254 nm), and photographs of the luminescent samples were taken under UV illumination. The results are presented in Figure 10.

**Figure 11:**
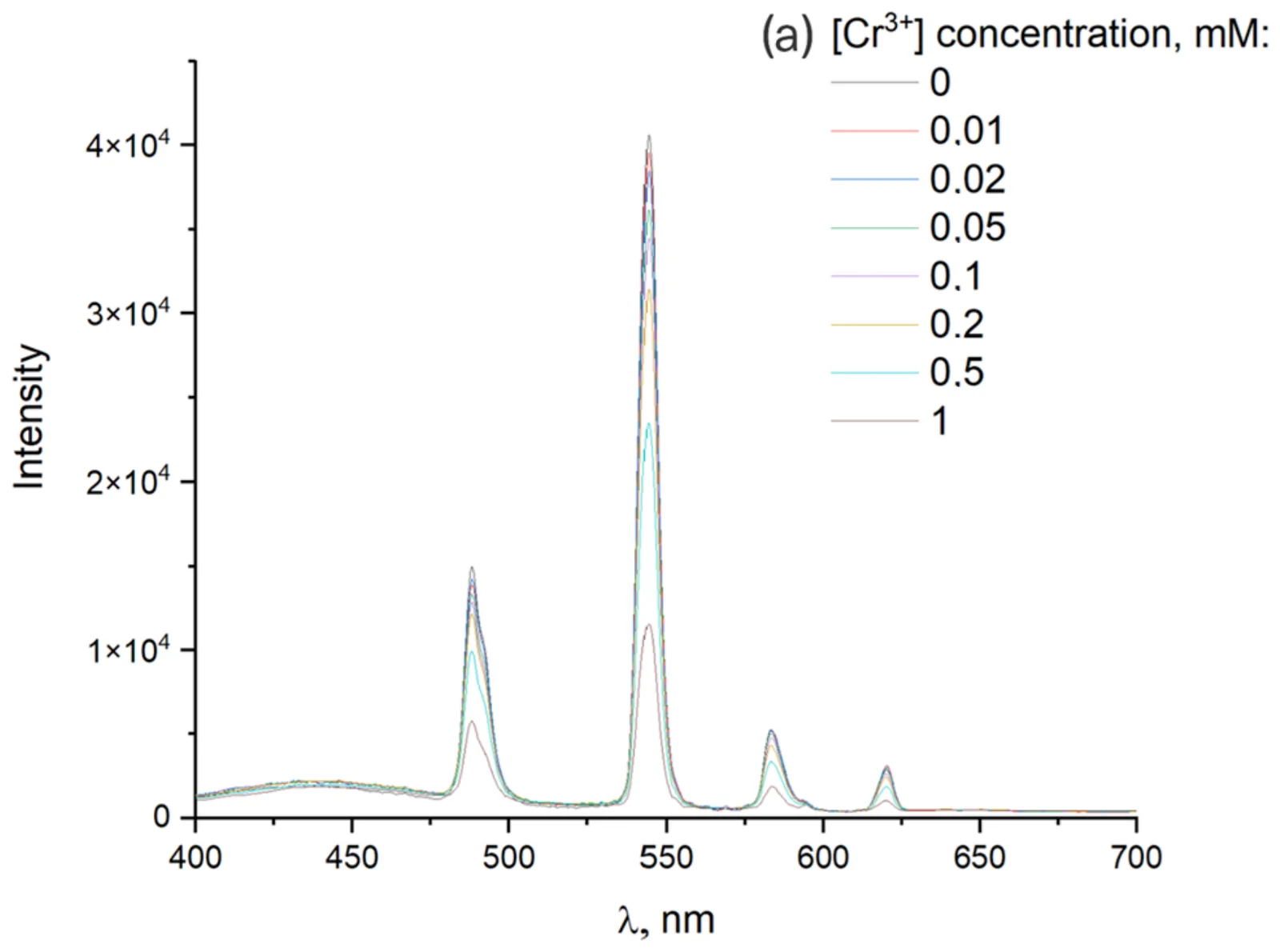

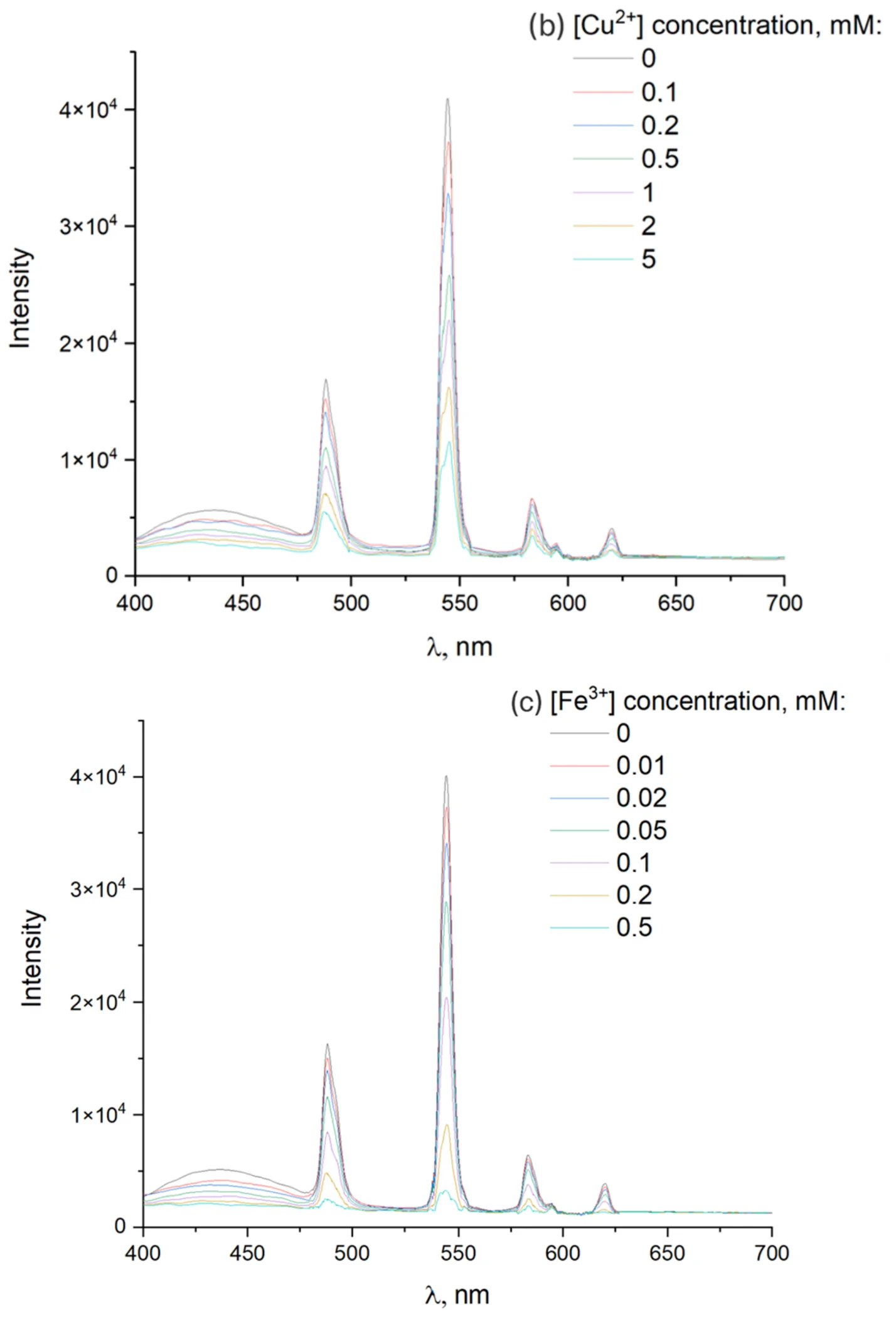
Emission spectra of (Tb_0.5_Gd_0.5_)_2_(Cl-1,4-bdc)_3_·5H_2_O with varying concentrations of added Cr^3+^ (**a**), Cu^2+^ (**b**), and Fe^3+^ (**c**) ions.

To study the sensing behaviour in more detail, luminescence spectra of terbium chloroterephthalate suspensions were recorded upon the addition of various concentrations of Cr^3+^, Fe^3+^, and Cu^2+^ ions. For this purpose, a suspension of (Tb_0.5_Gd_0.5_)_2_(Cl-1,4-bdc)_3_·5H_2_O with a concentration of 0.5 mg/mL was prepared. Microliter volumes of metal salt solutions with concentrations in the range of 0.0001–0.1 M were successively added to 3 mL of the suspension in a quartz cuvette, yielding final quencher ion concentrations of 0.001–5 mM. The luminescence spectra of the suspensions were recorded using an excitation wavelength of 320 nm (Figure 11).

A noticeable decrease in luminescence intensity for Cr^3+^ and Fe^3+^ was observed at a concentration of 0.01 mM. Cu^2+^ ions exhibited the lowest quenching efficiency, with significant quenching observed only at 0.1 mM. Emission spectra of (Tb_0.5_Gd_0.5_)_2_(Cl-1,4-bdc)_3_·5H_2_O with varying concentrations of added Cr^3+^, Cu^2+^, and Fe^3+^ ions are shown below.

**Figure 12:**
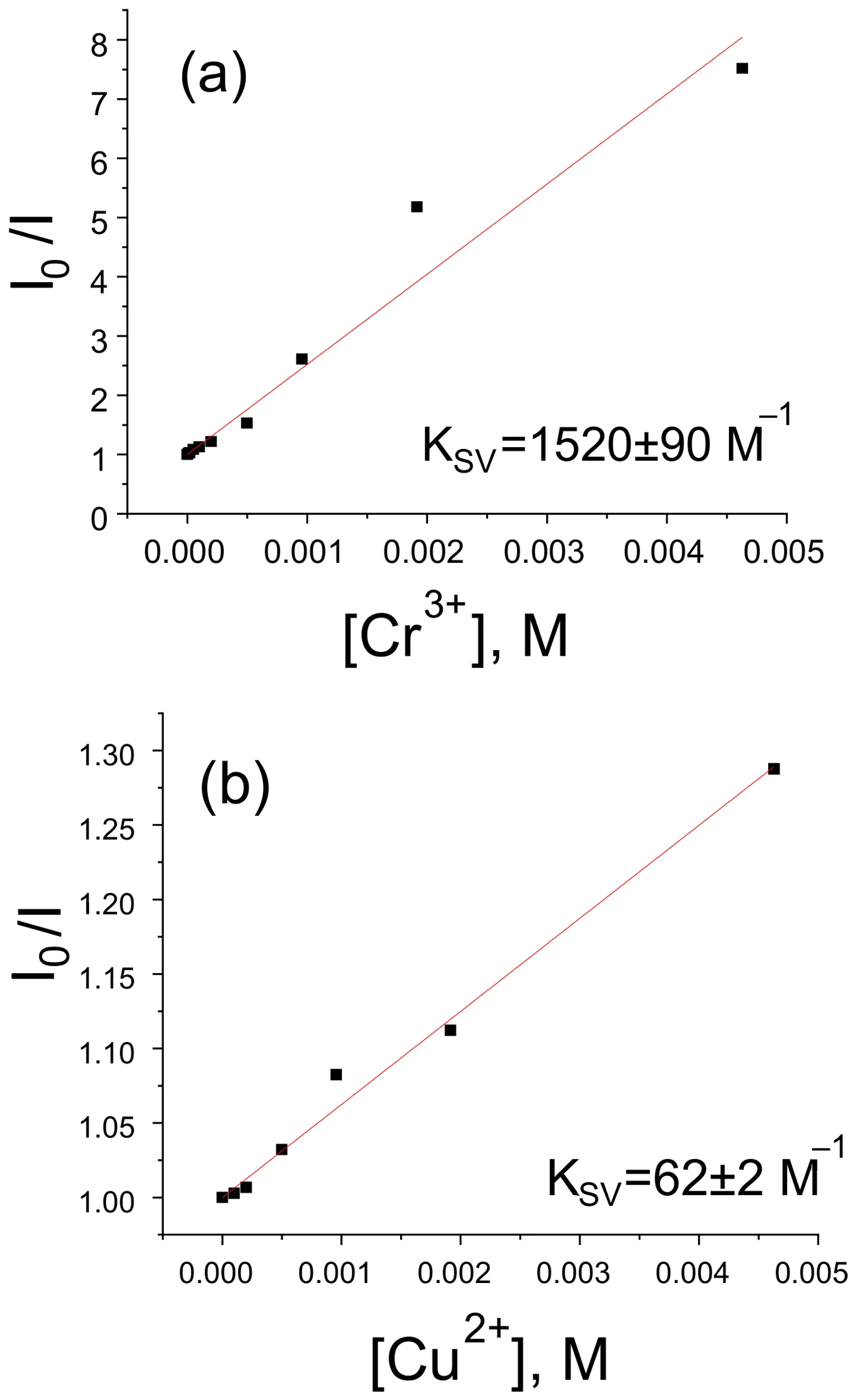

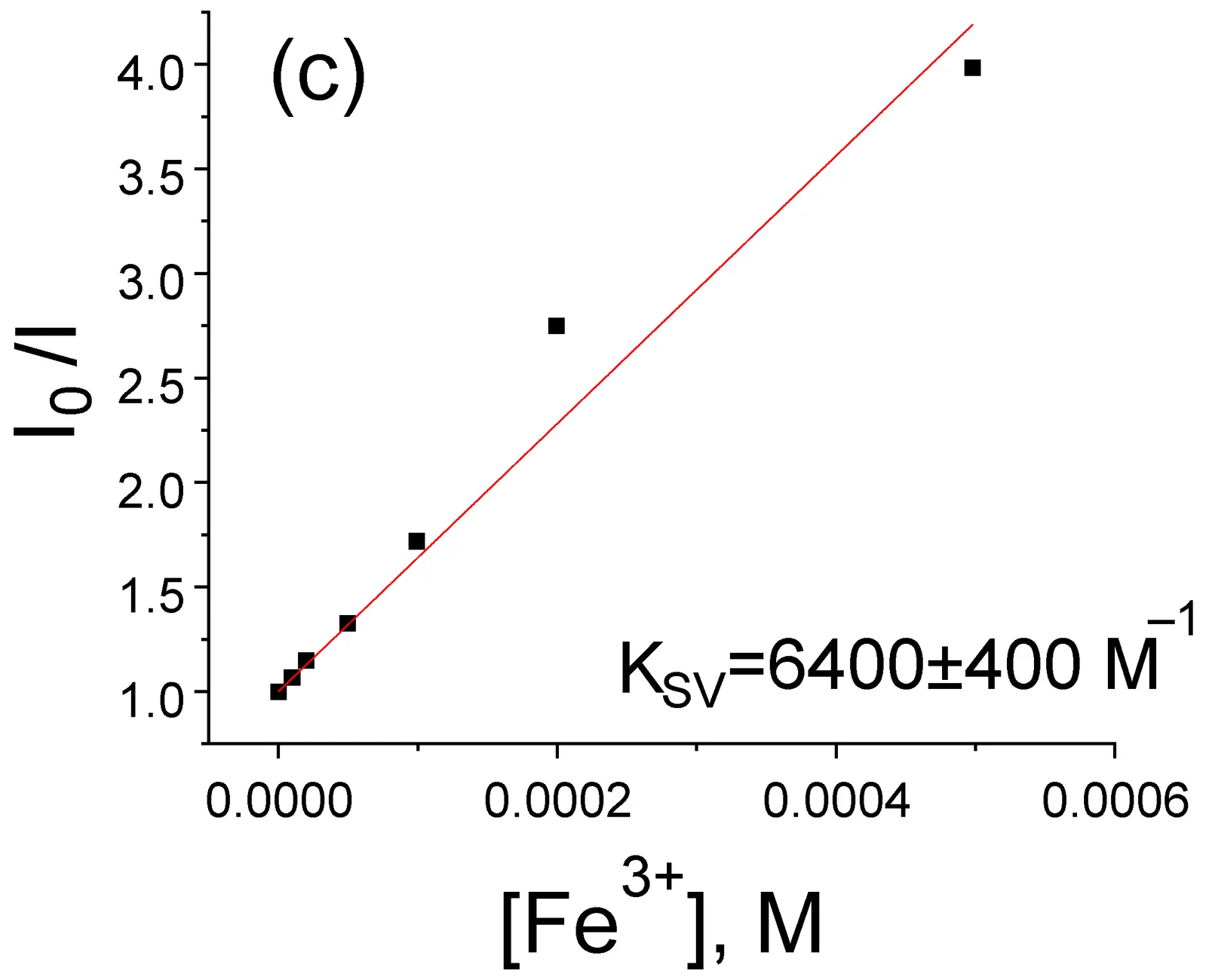
Dependence of I_0_/I (Tb_0.5_Gd_0.5_)_2_(Cl-1,4-bdc)_3_·5H_2_O on the concentration of Cr^3+^ (**a**), Cu^2+^ (**b**), and Fe^3+^ (**c**) ions for determining the Stern–Volmer constant.

To quantitatively assess the quenching efficiency, plots of integrated luminescence versus quencher ion concentration were plotted in *I*_0_/*I* vs. [M*^n^*^+^] coordinates, where *I* is the integrated luminescence intensity with the addition of a metal ion at [M*^n^*^+^] concentration, and *I*_0_ is the integrated luminescence intensity in the absence of the additive. These dependencies (Figure 12) were approximated by linear functions of the form

$$\frac{I_{0}}{I}=1+K_{SV}*[M^{n+}],$$

where *K_SV_* is the quenching constant (Stern–Volmer constant).

A comparative analysis of the K_SV_ values showed that Cr^3+^ and Fe^3+^ ions quench the luminescence of terbium chloroterephthalate more effectively. Although the detection limit (0.01 mM) is higher than that of some state-of-the-art luminescent sensors, the primary goal of this work was to demonstrate the feasibility of using these new MOFs as luminescent probes and to investigate the quenching mechanism. Further optimization of the material (e.g., by reducing particle size or surface functionalization) may improve the sensitivity.

### 3.2. Fingerprinting

**Figure 13:**
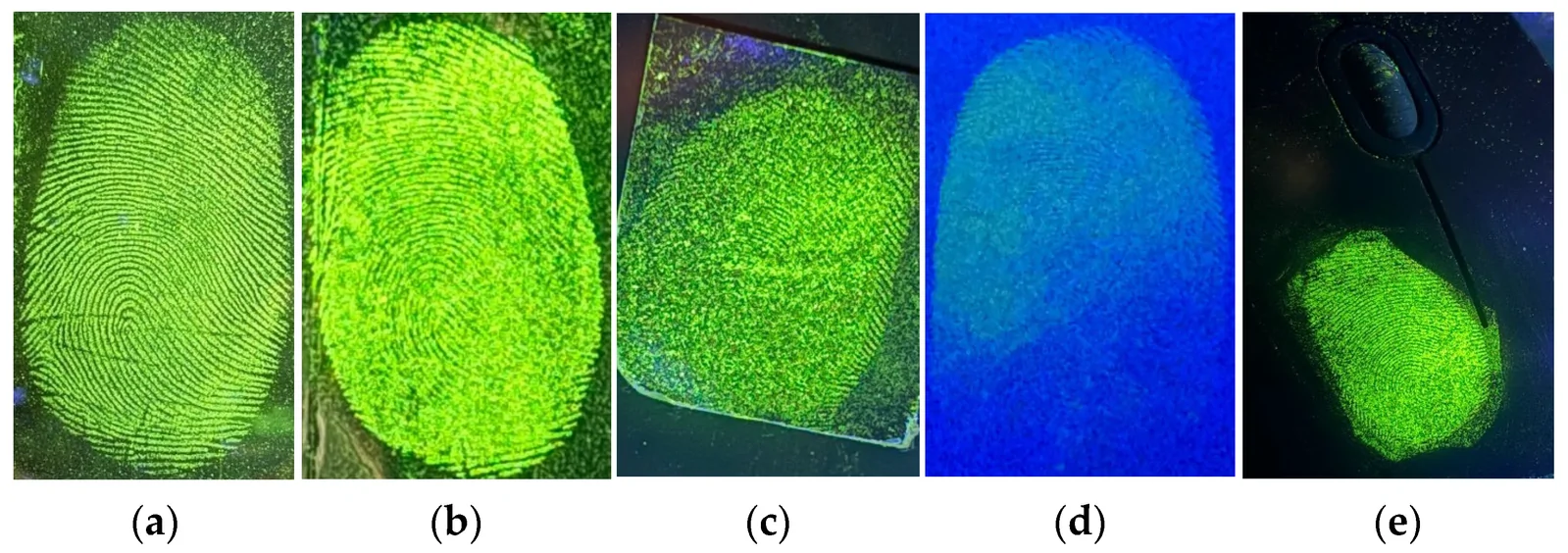
Luminescence images of latent fingerprints developed on transparent glass (**a**), aluminum foil (**b**), cardboard with a glossy surface (**c**), white paper (**d**), and computer mouse with a plastic case (**e**) with (Tb_0.5_Gd_0.5_)_2_(Cl-1,4-bdc)_3_·5H_2_O detected under the 254 nm illumination.

The capability of the synthesized compound for latent fingerprint development was tested on a range of surfaces. To prepare the latent fingerprints, the first author of this paper, Oleg Butorlin, as a volunteer, placed his right thumb onto the substrates without prior cleaning. Substrates included glass, aluminum foil, glossy cardboard, white paper, and the plastic body of a computer mouse. A fine layer of (Tb_0.5_Gd_0.5_)_2_(Cl-1,4-bdc)_3_·5H_2_O powder was then lightly distributed over each print with a feather brush; any surplus material was carefully swept away by gentle airflow from a blower. Imaging was performed under a 254 nm mercury vapour UV lamp, and a 500 nm long-pass filter was mounted in front of the camera to block the excitation light and enhance image contrast. Representative photographs are shown in Figure 13. Even on challenging backgrounds, with the exception of white paper, the method produced sharp, well-defined ridge patterns that could be discerned directly by eye, confirming the practical utility of these luminescent powders for fingerprint visualization.

**Figure 14:**
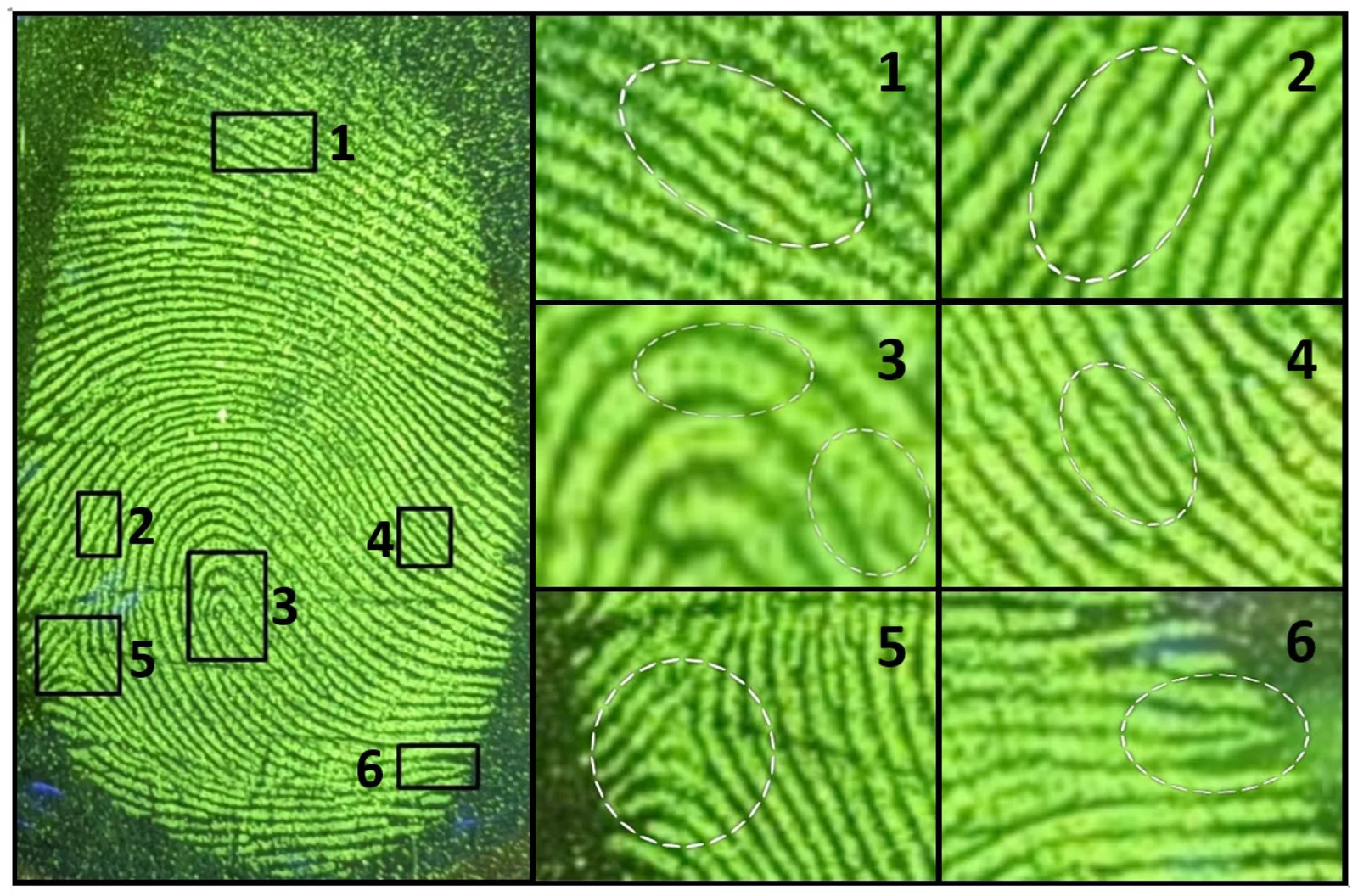
Luminescence images of latent fingerprints developed on transparent glass with (Tb_0.5_Gd_0.5_)_2_(Cl-1,4-bdc)_3_·5H_2_O detected under the 254 nm illumination in the dark field: the fine structure of latent fingerprint (in the dashed-line circle) bifurcation (1), spur (2), sweat pores (3) island (4), closed delta (5), loop (6).

Close-up views (Figure 14) reveal all the characteristic ridge minutiae needed for reliable identification. The high-magnification luminescence images clearly display fine structural details: bifurcation (1), spur (2), sweat pores (3), island (4), closed delta (5), and loop (6). In contrast to conventional carbon-based powders—which often smudge fine features, stain the surrounding substrate, and become nearly invisible on dark surfaces—our formulation strongly highlights the fingerprint ridges without contaminating the background. The powder adheres selectively to the papillary residues, delivering excellent contrast, straightforward application, and faithful preservation of ridge detail, thereby substantially increasing the confidence and accuracy of forensic analyses.

## 4. Materials and Methods

Terbium (III) chloride hexahydrate and gadolinium (III) chloride hexahydrate were purchased from Chemcraft (Kaliningrad, Russia); 2-chlorobenzene-1,4-dicarboxylic (chloroterephtalic, H_2_(Cl-1,4-bdc)) acid (>98%), and sodium hydroxide (>99%) were purchased from Sigma-Aldrich Chemie GmbH (Taufkirchen, Germany) and used without additional purification. The heterometallic terephthalate compounds with a general formula of (Tb_x_Gd_1−x_)_2_(Cl-1,4-bdc)_3_·5H_2_O were prepared by mixing 0.2 M TbCl_3_ and 0.2 M GdCl_3_ with 2 mL of 0.3 M Na_2_(Cl-1,4-bdc) aqueous solution followed by ultrasonic treatment for 10 min. The Na_2_(Cl-1,4-bdc) aqueous solution was prepared by dissolving of the H_2_(Cl-1,4-bdc) in sodium hydroxide aqueous solution. The TbCl_3_ and GdCl_3_ solutions were mixed in stoichiometric amounts, and the total volume of the solutions was 1 mL The resulting white precipitates were separated from the mixture using centrifugation at 4000× *g* and washed three times with deionized water. The samples were then dried at 60 °C for 48 h. The Tb^3+^/Gd^3+^ ratios in the heterometallic terephthalates were confirmed using energy dispersive X-ray spectroscopy (EDX) (EDX spectrometer EDX-800P, Shimadzu, Kyoto, Japan). The Tb/Gd ratios obtained from EDX were consistent with the expected ratios of Tb^3+^/Gd^3+^ within 1 at.% accuracy. Powder X-ray diffraction (PXRD) measurements were performed with a D2 Phaser (Bruker, Billerica, MA, USA) X-ray diffractometer using Cu Kα radiation (λ = 1.54056 Å). SEM images were obtained using a scanning electron microscope Zeiss Merlin (Carl Zeiss AG, Jena, Germany). Thermogravimetry curves were obtained using a TG 209 F1 Libra thermo-microbalance (Netzsch, Hanau, Germany). Elemental (CHN) analysis was performed using the LECO TruSpec MICRO Elemental Analyzer (LECO Corporation, St. Joseph, MI, USA). The measurement of FTIR spectra were recorded using IRAffinity-1 spectrometer (Shimadzu, Kyoto, Japan). To carry out photoluminescence studies, the synthesized samples (20 mg) and potassium bromide (300 mg) were pressed into pellets (diameter 13 mm). The photoluminescence data were obtained with a Fluoromax-4 fluorescence spectrometer (Horiba Jobin Yvon, Kyoto, Japan) in perpendicular geometry. Lifetime measurements were performed with the same spectrometer using a pulsed Xe lamp (pulse duration: 3 μs) in a timescale of 50 μs–10 ms. The absolute values of the photoluminescence quantum yields were recorded using a Fluorolog-3 Quantaphi-2 (Horiba Scientific, Kyoto, Japan) device using an integration sphere. All measurements were performed at room temperature.

## 5. Conclusions

In this work, a series of heterometallic rare-earth chloroterephthalate metal–organic frameworks (MOFs) with the general formula (Tb_x_Gd_1−x_)_2_(Cl-1,4-bdc)_3_·5H_2_O was successfully synthesized and comprehensively characterized. The systematic investigation revealed several key findings. All obtained compounds form isostructural solid solutions across the entire composition range, as confirmed by XRD, TGA, IR spectroscopy, and EDX analysis. The synthesized MOFs exhibit bright photoluminescence under UV excitation due to an efficient antenna effect from the chlorosubstituted terephthalate ligand. The photoluminescence quantum yield shows a non-monotonic dependence on the terbium(III) concentration, reaching a maximum of 71 ± 1% for the composition (Tb_0.5_Gd_0.5_)_2_(Cl-1,4-bdc)_3_·5H_2_O. The luminescence enhancement in chloroterephthalates is explained by elimination of Tb^3+^ concentrational quenching by the partial substitution of luminescent Tb^3+^ ions with optically inactive Gd^3+^ ones without altering the crystalline structure. Screening against ten different ionic species showed that the luminescence of the MOF is significantly quenched by Cr^3+^, Fe^3+^, and Cu^2+^. The Stern–Volmer study confirmed that terbium chloroterephthalate is particularly sensitive to Cr^3+^ and Fe^3+^, with detection limits of 0.01 mM. These MOFs are therefore promising candidates for the selective luminescent detection of chromium and iron ions. The materials exhibit a distinctive particle morphology (hourglass-shaped aggregates of plate-like particles) and high thermal stability. The combination of high quantum yields, pure colour emission, and stability makes these heterometallic chloroterephthalate MOFs a promising new class of functional phosphors. The most immediate practical application lies in their use as highly effective luminescent fingerprint powders for forensic science, offering superior contrast, selectivity, and signal stability on various surfaces.

## Figures and Tables

**Table 1:** Results of energy dispersive analysis (EDX).

χ (Tb), %	5	10	20	30	40	50	60	70	80	90
Tb	6 ± 1	11 ± 1	21 ± 1	32 ± 2	42 ± 2	55 ± 3	64 ± 4	75 ± 4	83 ± 5	93 ± 5
Gd	94 ± 5	89 ± 5	79 ± 4	68 ± 4	58 ± 3	45 ± 2	36 ± 2	25 ± 1	17 ± 1	7 ± 1

**Table 2:** Results of elemental analysis.

Compound Formula	ω(C)calc., %	ω(C)exp., %	ω(H)calc., %	ω(H)exp., %
Gd_2_(Cl-1,4-bdc)_3_·5H_2_O	28.82	28.61	1.91	1.82
Tb_2_(Cl-1,4-bdc)_3_·5H_2_O	28.72	28.59	1.91	1.92

**Table 3:** Lifetimes (τ) and photoluminescence quantum yields PLQY of (Tb_x_Gd_1−x_)_2_(Cl-1,4-bdc)_3_∙5H_2_O.

χ (Tb), %	τ, ms	PLQY, %
0.1	1.14 ± 0.01	3 ± 1
1	1.15 ± 0.01	17 ± 1
5	1.16 ± 0.01	31 ± 1
10	1.14 ± 0.01	44 ± 1
20	1.13 ± 0.01	63 ± 1
30	1.12 ± 0.01	62 ± 1
40	1.10 ± 0.01	66 ± 1
50	1.09 ± 0.01	71 ± 1
60	1.07 ± 0.01	66 ± 1
70	1.05 ± 0.01	57 ± 1
80	1.02 ± 0.01	58 ± 1
90	0.99 ± 0.01	54 ± 1
100	0.92 ± 0.01	41 ± 1

## Data Availability

The data presented in this study are available on request from the corresponding author.
